# Ethnic Differences in Peripheral Skeletal Development Among Urban South African Adolescents: A Ten‐Year Longitudinal pQCT Study

**DOI:** 10.1002/jbmr.3279

**Published:** 2017-10-09

**Authors:** Simon M Schoenbuchner, John M Pettifor, Shane A Norris, Lisa K Micklesfield, Ann Prentice, Kate A Ward

**Affiliations:** ^1^ Medical Research Council (MRC) Elsie Widdowson Laboratory Cambridge UK; ^2^ South African Medical Research Council (SAMRC)/Wits Developmental Pathways for Health Research Unit, Department of Paediatrics, School of Clinical Medicine University of the Witwatersrand Johannesburg South Africa; ^3^ Medical Research Council (MRC) Lifecourse Epidemiology Unit University of Southampton Southampton UK

**Keywords:** BONE QCT, EPIDEMIOLOGY, GENERAL POPULATION STUDIES

## Abstract

There are no longitudinal pQCT data of bone growth and development from sub‐Saharan Africa, where rapid environmental, societal, and economic transitions are occurring, and where fracture rates are predicted to rise. The aim of this study was to compare skeletal development in black and white South African adolescents using longitudinal data from the Birth to Twenty study. The Birth to Twenty Bone Health subcohort consisted of 543 adolescents (261 [178 black] girls, 282 [201 black] boys). Annual pQCT measurements of the radial and tibial metaphysis and diaphysis were obtained between ages 12 and 22 years (distal metaphysis: cross‐sectional area [CSA] and trabecular bone mineral density [BMD]; diaphysis: total and cortical CSA, cortical BMD, and polar stress‐strain index [SSIp]). Age at peak height velocity (APHV) was calculated to account for differences in maturational timing between ethnic groups and sexes. Mixed‐effects models were used to describe trajectories for each pQCT outcome. Likelihood‐ratio tests were used to summarize the overall difference in trajectories between black and white participants within each sex. APHV (mean ± SD years) was similar in black (11.8 ± 0.8) and white (12.2 ± 1.0) girls, but delayed in black (14.2 ± 1.0) relative to white boys (13.3 ± 0.8). By 4 years post‐APHV, white adolescents had significantly greater cortical CSA and SSIp than black adolescents at the radius. There were no significant differences at the radial metaphysis but there was some divergence, such that black adolescents had greater radial trabecular BMD by the end of follow‐up. At the tibia, white adolescents had lower diaphyseal CSA and SSIp, and greater metaphyseal CSA. There was no ethnic difference in tibial trabecular BMD. There are ethnic differences in bone growth and development, independent of maturation, in South African adolescents. This work gives new insights into the possible etiology of childhood fractures, which occur most commonly as peripheral sites. © 2017 The Authors. *Journal of Bone and Mineral Research* Published by Wiley Periodicals Inc.

## Introduction

Growth and development of the skeleton are important determinants of subsequent bone health in adulthood. Comparisons of musculoskeletal phenotype in children between groups with different adult fracture risk may help define the structural determinants of bone health across the lifecourse.^1^ The Bone Health subcohort of the Birth‐to‐Twenty (Bt20) cohort provides an opportunity to address this with longitudinal peripheral quantitative computed tomography (pQCT) measures of bone in black and white adolescents from Johannesburg, South Africa. In this cohort, cross‐sectional results from an early follow‐up showed that at 13 years of age, black adolescents of both sexes had wider tibial diaphyses (shafts) than white adolescents, and black boys also had greater cortical bone mineral density (BMD) than white boys.^2^ White adolescents were also more likely than black children to have sustained a fracture by the age of 18 years.^3^, ^4^ It has also been shown that there were ethnic differences in age at menarche and skeletal maturation in Bt20, which may contribute to differences in peripheral skeletal development.^5^, ^6^, ^7^, ^8^, ^9^, ^10^, ^11^ Skeletal maturation in black males was delayed by about 6 months compared to their white peers.^9^ Skeletal differences in childhood were similar to those described from DXA‐derived areal BMD and hip structural analysis outcomes in postmenopausal South African and US women.^12^ Similarly, hip fracture incidence was reported to be much lower in sub‐Saharan Africa populations compared to those in the United States and Scandinavia.^13^, ^14^ In contrast, there was no evidence of ethnic differences in vertebral fracture prevalence among South African women.^15^

The number of people at risk of osteoporotic fracture is predicted to double by 2040, with demographic changes shifting much of this burden to low‐income and middle‐income countries,^16^ but there is a lack of prospective fracture data from sub‐Saharan Africa, and no longitudinal studies linking changes in bone structure or mineral density during childhood and adolescence to peak bone mass. Existing studies from other low‐income and middle‐income countries include the New Delhi Birth Cohort, which found associations between infant growth and peak bone mass, and between BMI gain during childhood and adolescence and peak bone density.^17^ Elsewhere prospective DXA data have been collected in the Bone Mineral Density in Childhood Study (US) and Saskatchewan Pediatric Bone Mineral Accrual Study (Canada).^18^, ^19^ In the University of British Columbia Healthy Bone Study, prospective measures of pQCT and high resolution pQCT (HRpQCT) were collected and have recently been reported.^20^, ^21^ Using longitudinal growth data from the UK National Survey of Health and Development, the timing of pubertal growth (determined either by age at menarche, voice breaking, or by Super Imposition by Translation and Rotation [SITAR]) and height and weight growth patterns were related to bone health in early old‐age (60 to 64 years).^22^, ^23^, ^24^ Together, these studies have shown that the amount of longitudinal growth, weight change, and bone mineral accrual during adolescence are important predictors of adulthood bone health and future risk of osteoporotic fracture.^25^, ^26^

We therefore aimed to understand how ethnic differences in pQCT measurements track or change during adolescence in South African black and white boys and girls, and hence whether previously described differences in early adolescence were retained into early adulthood, and whether those differences were independent of maturational differences between ethnic groups.

## Subjects and Methods

### Participants

The Birth‐to‐Twenty study is the largest and longest‐running birth cohort in Africa.^27^ Pregnant women were approached in public antenatal clinics in Soweto, Johannesburg, in late 1989, with the aim of recruiting all singleton births during a 7‐week period from April to June 1990. To be eligible for the study, mothers and infants had to be continuously resident in the Soweto area for at least the first 6 months after birth. Total enrollment was 3273 children. A wide variety of outcomes was measured, covering growth, nutrition, healthcare access, socioeconomic status, and educational progress.^27^, ^28^

A specific theme on bone health was added at the age of 9 years, when a subset of black and white children from the original cohort was enrolled in the Bone Health subcohort. The terminology follows “racial” classifications that were imposed under the apartheid regime until 1994, and continue to be used in South Africa today. The term ethnicity is used to include the socioeconomic consequences of this classification as well as assumptions of common ancestry. White children were underrepresented in the original cohort because white families at the time were more likely to use private health facilities. In addition, there had been greater sample attrition among white children during the first 10 years of the study. For these reasons, an additional sample of 120 white children, all born during the original recruitment period, was recruited into the Bone Health subcohort in 2000.^27^ The time points of data‐collection do not necessarily correspond to the chronological age of the cohort and for this reason time points will be referred to as year 10 (Y10), year 13 (Y13), etc.

In total, 683 participants were included in the Bone Health subcohort. DXA and hand‐wrist radiography were performed annually from Y10 of the study,^9^, ^29^ and fractures were recorded retrospectively at time points Y10, Y13, Y15, and Y17/Y18.^3^, ^4^ pQCT was performed annually from Y13 onward, on 543 participants. Girls who were or had been pregnant were excluded. The pQCT measurements cover an age range of 12.3 to 22.2 years for boys, and 12.2 to 22.2 years for girls, with a median of six scans per individual.

From the combined sample of the original Bt20 cohort with the additional participants recruited into the Bone Health subcohort, height measurements were obtained for 2522 individuals. Table 1 shows participant numbers in the Bone Health subcohort by ethnic group, together with the percentage in each group that was still present during the final 2 years of follow‐up (Y19 and Y20).

**Table 1:** Participant Numbers in the Bone Health Subcohort

Sex	Ethnicity	Participants (sample retention) *n* (%)^a^	Visits per participant median (IQR)
Girls	Black	178 (80)	7 (6, 7)
	White	83 (60)	6 (4, 6)
Boys	Black	201 (85)	7 (6, 7)
	White	81 (65)	5 (3, 6)
Total		543 (77)	6 (5, 7)

IQR = interquartile range.

^a^ Sample retention (%) until at least study year 19.

### Bone imaging

Cross‐sectional images of the radius and tibia were obtained using pQCT, performed using the Stratec XCT‐2000 (Stratec Medizintechnik, Pforzheim, Germany) scanner on the left leg and forearm. Two trained technicians acquired all scans throughout the study using the same machine (interoperator variation <1%). Limb length was measured using a tape measure between palpable bony landmarks. Leg length was measured while the participant was seated, with feet flat on the floor and the knees bent at 90 degrees, from the medial tibial plateau to the medial malleolus. Forearm length was measured with the arm bent at 90 degrees, from the olecranon to the ulnar styloid process. Scan locations were defined by a percentage of limb length, measured from a reference line that was positioned at the distal end plate of the relevant bone using a scout view scan. If epiphyseal fusion was incomplete, the reference line was positioned at the medial border of the growth plate instead. Following standard pQCT protocols,^30^ metaphyseal scans were obtained at the 4% radius and tibia, and diaphyseal scans at the 65% radius and 38% tibia with a slice thickness of 2.3 mm.

The voxel size for scans changed during the 10 years of the study: at the radial metaphysis, voxel sizes were 0.59 mm in Y12 to Y13, 0.4 mm in Y14 to Y16, and 0.5 mm in Y17 to Y20. At the radial diaphysis, voxel sizes were 0.4 mm in Y12 to Y16 and 0.5 mm in Y17 to Y20. At the tibia, a voxel size of 0.4 mm was used throughout the study, but a small number of scans each year were obtained at a voxel size of 0.5 mm. To correct for these changes, we added a categorical variable in the analysis models. We graphically compared the results from this pooled analysis to the results of separate models for each voxel size and they agreed well.

All images were processed by the same person (SMS) using the manufacturer's software (Stratec XCT 6.20; Stratec Medizintechnik), with thresholds of 710 mg/cm^3^ for cortical bone, 280 mg/cm^3^ for total bone and polar stress‐strain index (SSIp) at the diaphyseal sites (cortical mode 1), and 180 mg/cm^3^ for area and density at the metaphyseal sites (contour mode 1, peel mode 1). Given the large number of scans obtained, images were not all individually graded. Instead, ∼5% of images from each study year were randomly sampled for scrutiny, and extreme outliers were additionally checked. Outliers were excluded only if inspection of the images indicated incorrect positioning or excessive movement, but were otherwise retained.

Outcome measures were metaphyseal total cross‐sectional area (CSA) and trabecular bone mineral density (BMD, measured in the inner 45% of the metaphyseal cross‐section), and diaphyseal total CSA, cortical CSA, medullary CSA, cortical BMD, and SSIp at the proximal sites. SSIp is a composite measure representing the distribution of voxels containing bone from the central axis of the shaft, weighted by voxel density, estimating the whole bone's resistance to torsion. Short‐term coefficients of variation (CVs) for pQCT of the proximal tibia in adolescents are 0.6% for diaphyseal CSA, 3.0% for medullary CSA, 0.5% for cortical BMD, and 2.8% for SSIp.^30^

### Statistical methods

All analyses were performed using R version 3.3.2 (R Foundation for Statistical Computing, Vienna, Austria; https://www.r-project.org/)^31^ with the following packages: dplyr version 0.5.0 for data manipulation,^32^ lme4 version 1.1‐12 for mixed‐effects modeling,^33^ sitar version 1.0.3 for SITAR,^34^ and ggplot2 version 2.2.1 for graphics.^35^ Continuous variables are summarized as mean ± SD and regression coefficients are reported with 95% confidence intervals. When data were categorized by biological age (only in Table 2), summary statistics (mean ± SD) were weighted if a subject was observed more than once within a biological age group.

**Table 2:** Summary Statistics for the Bone Health Subcohort

		Black				White			
Sex	Biological age group^a^	*n*	Age (years)	HAZ^b^	BAZ^c^	*n*	Age (years)	HAZ^b^	BAZ^c^
Girls	1	157	13.2 ± 1.0	–0.26 ± 0.90	0.22 ± 1.19	67	13.8 ± 1.1	0.41 ± 1.20	0.15 ± 1.08
	2	165	14.2 ± 0.9	–0.41 ± 0.83	0.34 ± 1.14	64	14.8 ± 1.1	0.39 ± 1.01	0.26 ± 1.10
	3	164	15.1 ± 0.9	–0.54 ± 0.82	0.44 ± 1.08	64	15.7 ± 1.2	0.29 ± 1.06	0.14 ± 1.01
	4	166	16.1 ± 1.0	–0.65 ± 0.86	0.48 ± 1.07	56	16.5 ± 1.1	0.25 ± 1.05	0.27 ± 0.98
	5	154	17.0 ± 0.9	–0.70 ± 0.88	0.51 ± 1.06	54	17.5 ± 1.1	0.20 ± 0.96	0.18 ± 1.05
	6	123	18.0 ± 0.9	–0.73 ± 0.87	0.64 ± 1.08	40	18.4 ± 1.1	0.34 ± 1.13	0.31 ± 1.12
	7	98	18.9 ± 0.8	–0.79 ± 0.90	0.62 ± 1.03	36	19.2 ± 1.0	0.39 ± 1.08	0.43 ± 1.14
Boys	0	185	14.5 ± 1.1	–0.55 ± 1.00	–0.18 ± 1.25	67	13.9 ± 0.9	0.56 ± 1.19	0.09 ± 1.15
	1	186	15.5 ± 1.1	–0.46 ± 0.90	–0.26 ± 1.18	58	14.7 ± 0.8	0.69 ± 1.10	0.16 ± 1.02
	2	175	16.5 ± 1.1	–0.52 ± 0.85	–0.35 ± 1.12	58	15.8 ± 0.8	0.53 ± 1.09	0.14 ± 1.09
	3	166	17.4 ± 1.0	–0.60 ± 0.88	–0.39 ± 1.13	57	16.8 ± 0.9	0.37 ± 1.08	0.21 ± 0.99
	4	140	18.3 ± 1.0	–0.73 ± 0.88	–0.41 ± 1.20	51	17.8 ± 0.8	0.29 ± 1.09	0.10 ± 1.00
	5	115	19.4 ± 1.1	–0.76 ± 0.93	–0.45 ± 1.01	31	18.5 ± 0.7	0.10 ± 1.06	0.36 ± 0.98
	6	75	20.3 ± 1.1	–0.85 ± 0.90	–0.38 ± 1.21	30	19.3 ± 0.8	0.00 ± 1.04	0.35 ± 1.01

^a^ Biological age group: completed years post‐APHV. Summary statistics were weighted if a subject was observed more than once within the biological age group.

^b^ HAZ = height‐for‐age *Z*‐scores.^57^

^c^ BAZ = BMI‐for‐age *Z*‐scores.^57^

Ethnic and sex differences in maturational timing (assessed by skeletal age) have previously been described in this cohort,^9^ so all models were corrected for this by using biological age (years post‐APHV) instead of chronological age. We used anthropometric data from all black and white participants in the Bt20 and Bone Health cohorts (*n* = 2522) to calculate individual estimates of APHV for each participant, using SITAR^34^ models within each sex and ethnic group. All Bt20 participants were included to improve the precision of the model by increasing sample size. The resulting participant‐specific estimates of APHV were used to define biological age, measured in years post‐APHV, for each member of the Bone Health subcohort. This definition of biological age corrects for known differences in maturational timing by offsetting an individual's age scale by a fixed amount, but it does not account for differences in the rate at which individuals pass through puberty; ie, 1 year of biological age has the same duration as 1 year of chronological age.^19^, ^20^, ^21^, ^36^

Trajectories for each pQCT outcome were modeled separately for each sex, using mixed‐effects models with biological age and ethnicity as predictors. Additional fixed effects were included for interactions between ethnicity and biological age, allowing the ethnic difference in growth rates to vary with biological age. Mixed‐effects models fit growth curves for all participants simultaneously, rather than separately to data from each participant, and they can handle missing data by using information from other individuals to inform the overall shape of the curve (subject to the assumption that observations are missing at random). It is assumed that some features of the growth curve (the fixed effects) are similar across all participants, whereas others (the random effects) can vary between individuals.

The form of the fixed effects, representing the average trajectory, was chosen by comparing a sequence of models of different complexity by biological age, either with no change, or linear, or as a natural cubic spline with up to four degrees of freedom. For each outcome, the model with the lowest Bayesian Information Criterion (BIC) is reported here. Random intercepts were included in all models, allowing for variation in the outcome between individuals. Random slopes were also included if this improved model fit (as assessed by BIC), allowing for variation in the rate of change of the outcome between individuals. Correlation between the intercepts and slopes indicates whether individuals with larger/denser bones also experienced faster growth.

The overall effect of the interaction terms was summarized by using a likelihood‐ratio test (LRT) to compare the chosen model to an equivalent model with all the interaction terms removed (the number of such terms depending on the degrees of freedom of the fixed effects). Results of the LRT are reported as χ^2^‐values, which were considered significant if *p* < 0.05. This summarizes the significance of the overall difference in the shape or slope of the trajectories between black and white participants within each sex.

To indicate how well each model explained variation in the outcome, the SD of the residuals (unexplained noise) was converted to a CV (ratio of the SD to the mean). This model‐implied CV can be interpreted as a measure of the long‐term precision of the measurements. The coefficients in most models were dependent on the centering of the biological age scale because of the interaction terms and random slopes. An arbitrary biological age of 4 years post‐APHV was therefore chosen to give interpretable coefficients. To give a visual summary the ethnic differences across the whole range of biological ages, and to indicate the magnitude of the differences on a common scale, the standardized effect size (Cohen's *d*, the ratio of the difference to the between‐individual standard deviation) was calculated for each biological age. This shows how the magnitude of the ethnic difference compares to the overall variability in the sample at that biological age: as a rule of thumb, *d* < 0.5 is considered a “small” effect and *d* > 0.8 a “large” effect.^37^

## Results

**Figure 1:**
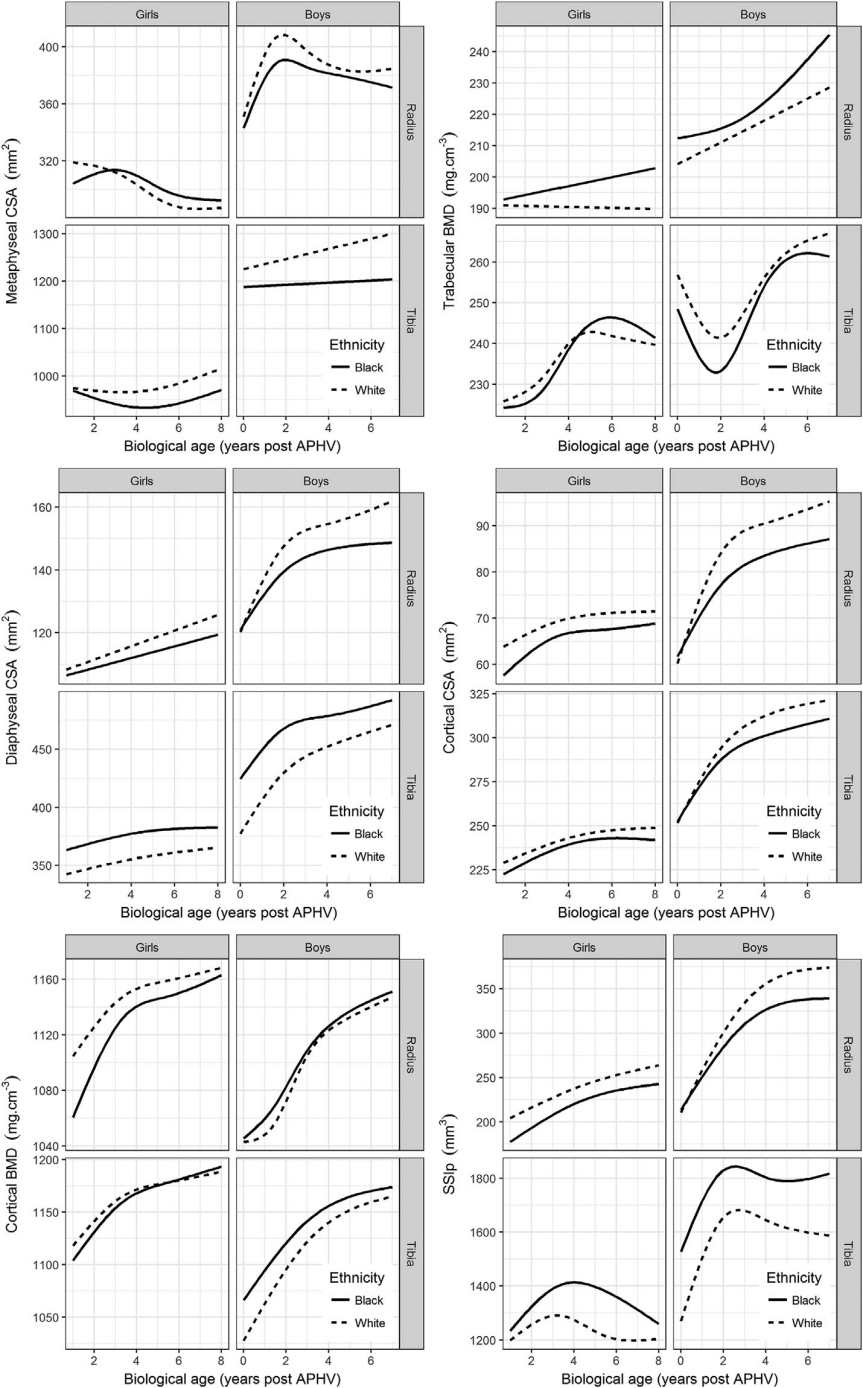
Mean trajectories of metaphyseal CSA (upper left panel), trabecular BMD (upper right panel), diaphyseal CSA (middle left panel), cortical CSA (middle right panel), cortical BMD (lower left panel), and SSIp (lower right panel) for black and white girls and boys at the radius and tibia, predicted based on the fixed effects coefficients in Table 3. All predictions were generated for a voxel size of 0.4 mm, with the random effects set to zero.

**Figure 2:**
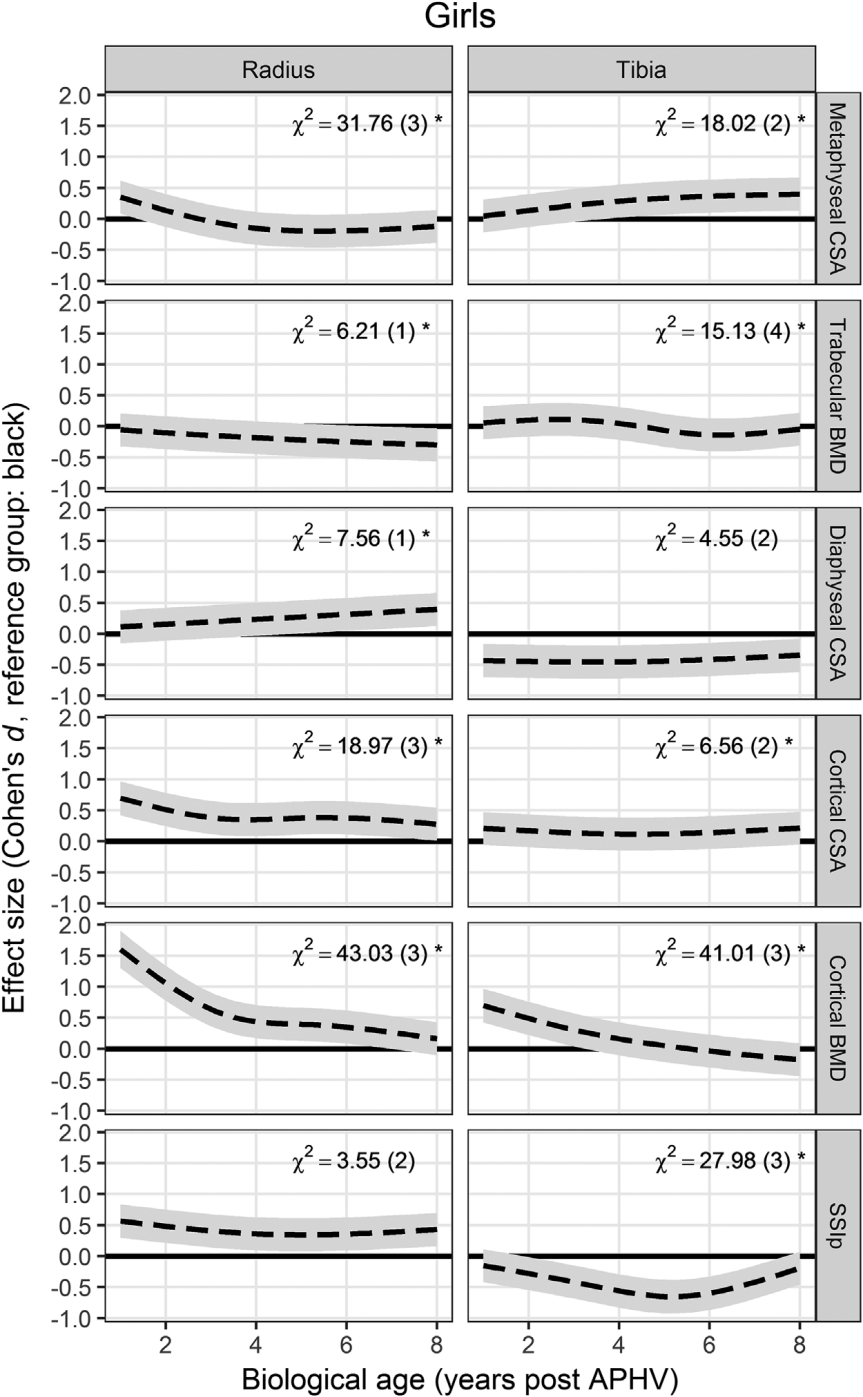
Ethnic differences among girls, expressed as effect sizes (Cohen's *d* = ratio of the mean difference to the pooled standard deviation), with pointwise 95% confidence bands. Interaction effects between ethnicity and biological age are represented by χ^2^ values, with (degrees of freedom) dependent on the number of spline terms fitted, *p < 0.05.

**Figure 3:**
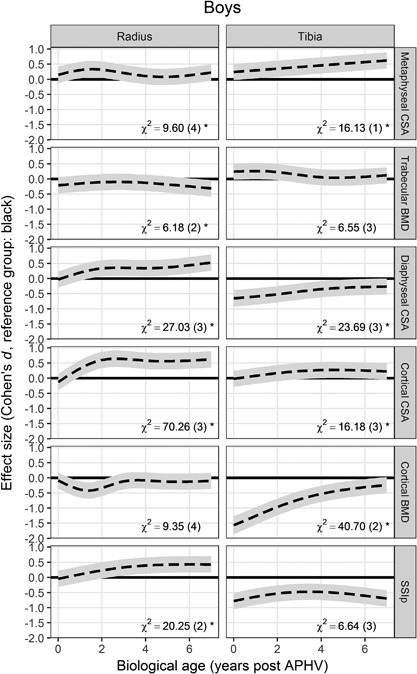
Ethnic differences among boys, expressed as effect sizes (Cohen's *d* = ratio of the mean difference to the pooled standard deviation), with pointwise 95% confidence bands. Interaction effects between ethnicity and biological age are represented by χ^2^ values, with (degrees of freedom) dependent on the number of spline terms fitted, *p < 0.05.

Summary statistics of participants are given in Table 2, grouped by biological age. Black participants of both sexes were short for their age and black boys were underweight, while white participants were at or above the WHO reference.^33^ Girls of the same biological age were also of similar chronological age. APHV (mean ± SD) was similar in black (11.8 ± 0.8 years) and white (12.2 ± 1.0 years) girls, but delayed among black boys (14.2 ± 1.0 years) relative to white boys (13.3 ± 0.8 years) Results of the mixed‐effects models are summarized in Table 3. No quantitative comparisons were made between boys and girls. Figure 1 shows the predicted growth curves for each outcome by site, sex, and ethnic group. Ethnic differences varied with biological age, as the two ethnic groups converged or diverged within each sex and as individuals within each group followed their individual trajectories. Figures 2 and 3 illustrate the estimated ethnic difference across the complete range of biological ages, expressed as effect sizes (Cohen's *d*; see final paragraph of Statistical methods), for girls and boys, respectively. Long‐term CVs are given in Table 3, using the calculation described in Statistical methods. The outcome with greatest precision was cortical BMD and that with worst precision was SSIp.

**Table 3:** Results From Mixed‐Effects Growth Models Summarizing Ethnic Differences in Peripheral Bone Development

				Fixed effects^a^		Random effects^b^			
Sex	Bone	Site	Outcome variable	Intercept (black)	Ethnicity (white‐black)	Intercept SD	Slope SD	Correlation	Residual SD (CV)^c^
Girls	Radius	4%	Meta CSA (mm^2^)	310 (304, 316)*	–6.37 (–17.44, 4.71)	39.9			15.0 (5%)
			Tb BMD (mg · cm^–3^)	197 (192, 202)*	–6.67 (–15.27, 1.93)	32.0	3.60	0.39	9.47 (5%)
		65%	Dia CSA (mm^2^)	112 (110, 114)*	3.74 (–0.28, 7.77)	14.8			6.04 (5%)
			Ct CSA (mm^2^)	66.8 (65.5, 68.1)*	3.18 (0.83, 5.52)*	8.70	0.335	0.32	1.87 (3%)
			Ct BMD (mg · cm^–3^)	1140 (1140, 1140)*	12.7 (5.7, 19.7)*	22.7	2.24	–0.50	15.4 (1%)
			SSIp (mm^3^)	220 (213, 227)*	17.3 (5.1, 29.5)*	43.7	1.76	0.68	17.8 (8%)
	Tibia	4%	Meta CSA (mm^2^)	934 (917, 950)*	32.9 (3.3, 62.5)*	108			37.4 (4%)
			Tb BMD (mg · cm^–3^)	238 (234, 243)*	1.44 (–5.84, 8.72)	26.4	3.08	0.30	6.86 (3%)
		38%	Dia CSA (mm^2^)	377 (370, 384)*	–22.0 (–34.6, –9.3)*	47.6	1.77	0.43	6.47 (2%)
			Ct CSA (mm^2^)	239 (235, 244)*	3.70 (–4.42, 11.81)	30.5	1.48	0.26	4.55 (2%)
			Ct BMD (mg · cm^–3^)	1170 (1170, 1170)*	3.63 (–1.69, 8.96)	19.6	2.43	–0.52	5.44 (0%)
			SSIp (mm^3^)	1410 (1380, 1450)*	–139 (–199, –78)*	214	20.6	–0.11	91.6 (6%)
Boys	Radius	4%	Meta CSA (mm^2^)	381 (373, 390)*	6.07 (–8.46, 20.59)	50.4	3.44	0.04	19.3 (5%)
			Tb BMD (mg · cm^–3^)	224 (218, 230)*	–5.73 (–16.28, 4.81)	37.2	5.25	0.60	12.8 (6%)
		65%	Dia CSA (mm^2^)	146 (143, 150)*	8.27 (1.89, 14.65)*	23.1	0.977	0.84	5.80 (4%)
			Ct CSA (mm^2^)	83.5 (81.8, 85.2)*	6.96 (3.89, 10.02)*	11.1	0.882	0.56	2.49 (3%)
			Ct BMD (mg · cm^–3^)	1130 (1120, 1130)*	–2.87 (–10.90, 5.15)	26.9	3.61	–0.50	13.2 (1%)
			SSIp (mm^3^)	326 (316, 337)*	29.1 (10.4, 47.8)*	66.4	5.68	0.78	23.4 (7%)
	Tibia	4%	Meta CSA (mm^2^)	1200 (1180, 1220)*	71.5 (31.7, 111.3)*	146			54.4 (5%)
			Tb BMD (mg · cm^–3^)	254 (249, 259)*	2.15 (–7.24, 11.53)	33.1	4.46	0.51	10.6 (4%)
		38%	Dia CSA (mm^2^)	479 (468, 489)*	–26.4 (–45.8, –7.1)*	71.1	5.01	0.65	14.5 (3%)
			Ct CSA (mm^2^)	301 (296, 306)*	11.2 (0.8, 21.7)*	38.3	3.81	0.60	7.11 (2%)
			Ct BMD (mg · cm^–3^)	1160 (1150, 1160)*	–15.6 (–22.1, –9.2)*	22.6	4.05	–0.58	10.0 (1%)
			SSIp (mm^3^)	1800 (1760, 1850)*	–158 (–246, –71)*	298			142 (8%)

BMD = bone mineral density; CSA = cross sectional area; SSIp = polar stress strain index; Meta = metaphyseal; Dia = diaphyseal; Tb = trabecular; Ct = cortical.

^a^ Fixed effects: Estimated with (95% confidence intervals), *p < 0.05. Spline terms for biological age are not shown, but the form of the modeled trajectories is illustrated in Fig. 1. The intercept is the mean value for black participants and the ethnicity coefficient is the difference of white from black participants, at an arbitrary biological age of 4 years post‐APHV.

^b^ Random effects: Between‐participant variation in intercepts and slopes, summarized as standard deviations (SDs). Where random slopes were included, their correlation with the random intercepts is also shown.

^c^ Residuals: Summarized as SD (in the units of the outcome variable) and as coefficients of variation (CV, %).

**Figure 4:**
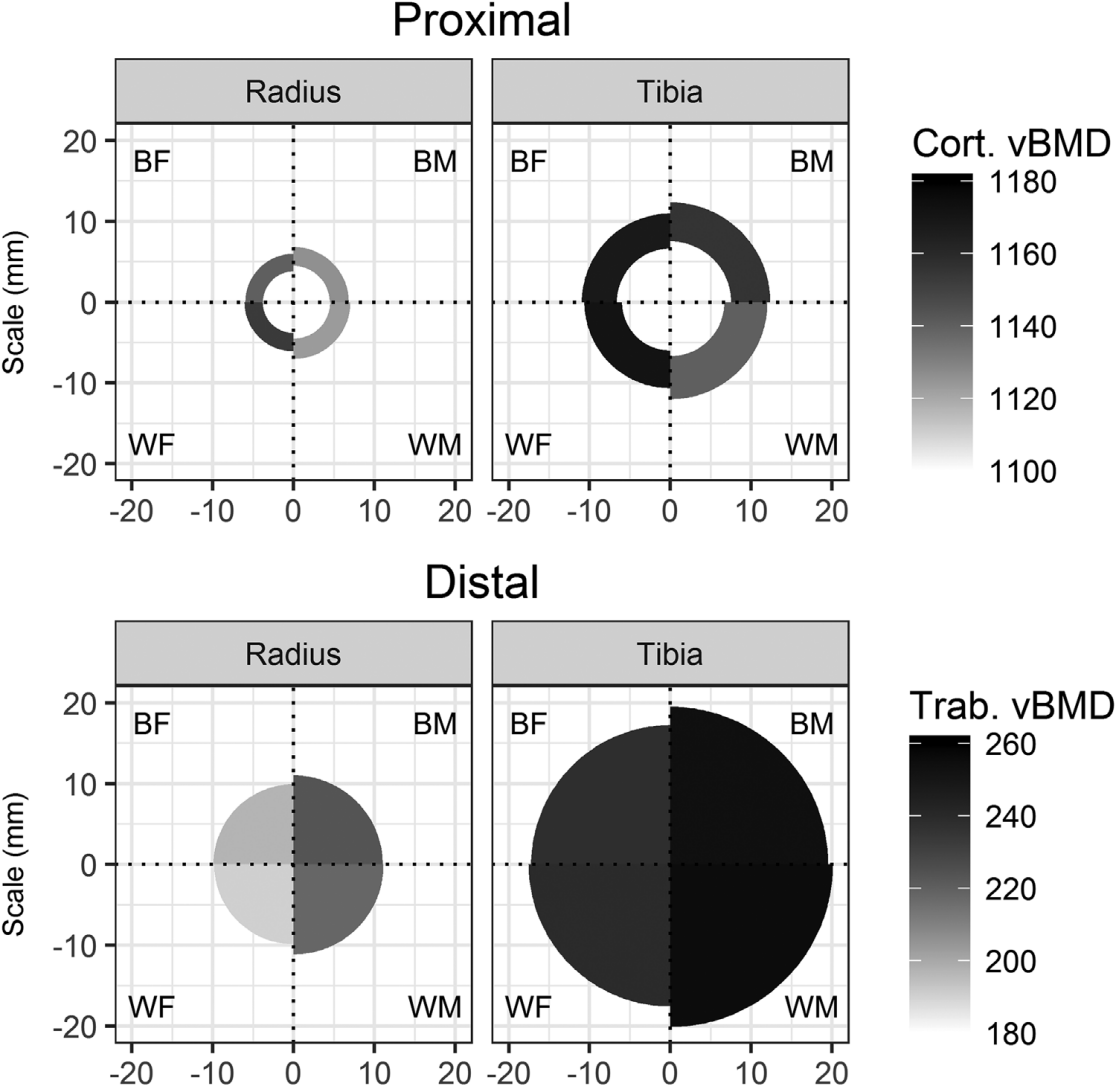
Schematic summary of pQCT outcomes at an arbitrary biological age of 4 years post‐APHV. Darker fill indicates greater cortical BMD (proximal site) or trabecular BMD (distal site), on distinct scales. Cross‐sectional areas are to scale but are drawn using an unrealistic cylindrical model of bone shape. Within each plot, the four quadrants show summaries for black boys (BM), white boys (WM), white girls (WF), and black girls (BF), clockwise from top right.

Figure 4 shows a schematic summary of the pQCT results at a biological age of 4 years post‐APHV; it is important to note this is an arbitrary biological age selected to provide a cross‐sectional snapshot of the longitudinal ethnic differences from the trajectories.

### Ethnic differences in metaphyseal outcomes in girls

Ethnic differences in radial metaphyseal CSA were not significant at most biological ages (Fig. 2). Girls in both ethnic groups showed some decrease in radial metaphyseal CSA from about 3 years post‐APHV (Fig. 1). Although trajectories did differ between ethnic groups (χ^2^, *p* < 0.05), there was no consistent pattern of convergence or divergence. At the tibia, the groups diverged (χ^2^, *p* < 0.05), resulting in greater metaphyseal CSA among white than among black girls, from about 3 years post‐APHV.

Trabecular BMD at the radius did not differ between ethnic groups until about 5 years post‐APHV (Fig. 2), but there was divergence (χ^2^, *p* < 0.05), leading to significantly lower trabecular BMD among white girls than black girls at later biological ages. There was no significant ethnic difference in trabecular BMD at the tibia at any biological age. Despite this, trajectories of change in trabecular BMD over the range of biological ages did differ, with the mean value of black girls overtaking that of white girls about 4 years post‐APHV (χ^2^, *p* < 0.05).

### Ethnic differences in diaphyseal outcomes in girls

White girls had similar diaphyseal CSA at the radius until about 4 years post‐APHV, but greater cortical CSA at all biological ages (Fig. 2). Diaphyseal CSA increased more quickly among white girls than among black girls (χ^2^, *p* < 0.05), leading to significant differences at later biological ages, while cortical CSA showed some convergence (Fig. 2). At the tibia, white girls had narrower bones at all biological ages, with no difference in cortical CSA. The mostly parallel trajectories of diaphyseal CSA between the groups were reflected by nonsignificant (NS) χ^2^ values.

Cortical BMD was initially higher among white girls, but the ethnic groups converged with biological age at both sites (Fig. 2). There was also convergence at the individual level, within groups: the negative correlation of the random effects (Table 3) shows that the increase in density at the radius and tibia was slower among girls who initially had greater cortical BMD. The ethnic difference remained significant at the radius until about 7 years post‐APHV, despite being small in absolute terms at only 12.7 mg/cm^3^ (∼1%) (Table 3). White girls consistently had higher SSIp at the radius, with near‐parallel trajectories (χ^2^, NS). At the tibia, SSIp was initially similar but diverged during adolescence, with white girls having significantly lower SSIp than black girls (Fig. 2). However, tibial SSIp then converged again. This difference in the shape of trajectories is reflected in the significant χ^2^ value. Despite increases in bone size and density, there were reductions in tibial SSIp from 3 or 4 years post‐APHV, particularly among black girls (Fig. 1).

### Ethnic differences in metaphyseal outcomes in boys

There were no significant ethnic differences in metaphyseal CSA of the radius at most biological ages (Fig. 3), although trajectories did differ between ethnic groups (χ^2^, *p* < 0.05). At the tibia, metaphyseal CSA of white boys was larger and grew more rapidly than that of black boys (χ^2^, *p* < 0.05).

Trabecular BMD was not significantly different between ethnic groups at the radius or at the tibia (Fig. 3). There was some divergence at the radius, where overall white boys had slightly lower trabecular BMD by the end of follow‐up. Boys in both ethnic groups showed a marked decline in tibial trabecular BMD during the first 2 years post‐APHV, but this was recovered in the subsequent 2 years (Fig. 1).

### Ethnic differences in diaphyseal outcomes in boys

White boys had significantly greater diaphyseal and cortical CSA at the radius after 1 year post‐APHV (Fig. 3). At the tibia, black boys had significantly greater diaphyseal CSA, but slightly smaller cortical CSA from about 3 years post‐APHV. The ethnicity‐age interactions were significant for both parameters (χ^2^, *p* < 0.05).

Cortical BMD was not significantly different between ethnic groups at the radius for most of the biological age range (Fig. 3). The ethnic difference was significant at the tibia but was very small in absolute terms (15 mg/cm^3^, ∼1%) (Table 3) and converged by the end of follow‐up (Fig. 3). Convergence at both sites between individuals within each group is indicated by the negative correlations of the random effects (Table 3). White boys had higher SSIp at the radius from about 2 years post‐APHV (χ^2^, *p* < 0.05), but consistently lower SSIp at the tibia (χ^2^, NS) (Fig. 3). There was some reduction in SSIp from about 3 years post‐APHV (Fig. 1), despite increases in bone size and density.

## Discussion

This is the first study to describe longitudinal bone growth and ethnic differences in density, geometry, and strength during adolescence in sub‐Saharan Africa. Ethnic differences in APHV were consistent with the results of previous analysis that assessed biological age using bone age scores from hand‐wrist radiographs from this cohort.^9^ By modeling height using SITAR to calculate APHV, we were able to adjust for known ethnic differences in timing of maturation and to show that there are persisting ethnic differences as the groups approach “peak bone mass.”

In general, black adolescents had larger (CSA), denser (cortical BMD), and stronger (SSIp) bones at the diaphyseal tibia. These results are broadly consistent with previous cross‐sectional findings in this cohort,^2^ illustrating that ethnic differences seen in early adolescence were sustained longitudinally into late adolescence. By adjusting for APHV, we have also confirmed that the previously observed cross‐sectional differences^2^ were not only a result of ethnic differences in maturational timing. The longitudinal modeling showed additional ethnic differences that emerged later in adolescence: black adolescents had smaller bones with similar density at the metaphyseal tibia; and smaller bones with smaller cortical CSA and lower strength (SSIp) at the diaphyseal radius. In contrast, they had slightly higher trabecular BMD at the metaphyseal radius, a common site of osteoporotic fracture. Another recent study in this cohort has shown that black adolescents had wider metacarpals, with thinner cortices than white adolescents,^38^ which broadly agrees with the longitudinal tibia rather than the radius data. The site‐specific differences show the importance of not extrapolating findings between skeletal sites.

Although data were collected at similar chronological ages, from 12 to 22 years, this translated into a biological age range of 0 to 7 years post‐APHV among boys, and 1 to 8 years post‐APHV among girls, so different ranges of biological ages were covered by the available pQCT observations. This sex difference in the range of biological ages covered in the study reflected the more advanced development of girls at any given chronological age during adolescence.

### Metaphyseal sites

We have shown a decline in radial metaphyseal CSA in boys and girls from about 3 years post‐APHV. This may represent the continued remodeling of bone after longitudinal growth has ceased, where the newly formed bone at the metaphysis is remodeled into the cortical shaft to create the characteristic long bone shape; this process is also known as inwaisting.^39^, ^40^ The scan location was defined as a percentage of bone length, but the decline in CSA occurs more than 2 years post‐APHV (Fig. 1), by which time longitudinal growth, and therefore growth in long bone length, has almost ceased and is unlikely to be due to imprecision of relocating scan sites, although this cannot be ruled out. The large long‐term precision error indicates this may be the case. Gabel and colleagues^21^ used HRpQCT to assess bone growth and showed less/no decline in CSA at later stages of maturity than in the current study; any changes were less than the precision error of the scanner.^41^ The differences in findings may be due to the use of HRpQCT in the Gabel study, measuring a volume of bone rather than a single slice (90 mm compared to 2.3 mm depth). This would mean the area is averaged across the 90‐mm slice, and would vary less than when relocating a single slice for measurement.

In girls, there were no changes in trabecular BMD during adolescent growth, but radius trabecular BMD was lower in white than in black girls. In contrast, there was early decline in tibial trabecular BMD among boys which may reflect increasing separation of the trabeculae during widening of the bone, so that a greater proportion of the area measured consisted of marrow rather than bone (Fig. 1). The differences in pattern between the sexes in this study are similar to those reported recently.^21^ Trabecular BMD depends on a combination of bone mineralization and trabecular thickness, but unlike HRpQCT the relative contributions of these factors cannot be captured by single‐slice pQCT. Previously published HRpQCT data^21^ indicated that continued modeling of the trabecular bone leads to a reduction in trabecular number as bone matures, together with an associated increase in trabecular thickness, more so in boys than in girls, which would explain the observed patterns in trabecular BMD in the current study of South African adolescents.

### Diaphyseal sites

Girls’ trajectories of diaphyseal and cortical CSA did not show the same rapid growth as boys in early adolescence, although this may in part be because the data began 1 year post‐APHV. There was expansion of the medullary cavity (ie, endocortical resorption) among white boys at both sites, among white girls at the radius only, and among black boys at the radius only. White girls generally had larger diaphyseal and cortical CSA at the radius than black girls. At the tibia, diaphyseal CSA was greater among black girls and there was no difference in cortical CSA, indicating larger bones with thinner cortices, with mostly parallel trajectories reflected by the nonsignificant χ^2^ values. At the tibia, white girls and boys had smaller diaphyseal CSA with greater cortical CSA, ie, narrower bones with thicker cortices and smaller medullary cross‐section, compared with black girls and boys. Previous longitudinal studies have offered conflicting evidence about medullary expansion in adolescence, indicating either no change, expansion, or contraction of the endocortical surface.^20^, ^42^, ^43^ Here the differences seem to be site‐specific.

Cortical BMD of the tibia was significantly greater in black than in white boys, which is consistent with previous reports.^44^ There are several possible explanations for this: for example, reduced porosity, increased mineralization, or lower bone turnover could contribute to greater cortical BMD.^45^ An additional contribution to changes or differences in cortical BMD may come from a technical artifact: as the cortex thickens, a smaller proportion of voxels lies on the boundary between cortical bone and soft tissue. This would reduce the influence of the partial volume effect, which contributes to underestimation of cortical BMD in small bones.^46^, ^47^

Earlier work in the Bone Health subcohort reported differences in childhood fracture rates between black and white children, with lower risk in the former.^3^ The greater tibial SSIp of black South African girls and boys is consistent with this observation. However, the shape of the trajectory is unexpected: tibial SSIp declined from about 4 years post‐APHV among girls and, to a lesser extent, from 3 years post‐APHV among boys. This was a period of slow growth in diaphyseal CSA and cortical BMD, with little endocortical resorption (except in white boys). Reduced SSIp may reflect a redistribution of bone within the cortex: voxels further from the central axis of the bone, which would normally contribute more to SSIp, may be under‐mineralized compared to those closer to the center, but the addition of new bone at the periosteal surface should increase it. The ethnic difference in SSIp is reversed at the radius, in a direction consistent with differences in diaphyseal CSA and cortical BMD, but inconsistent with differences in fracture risk. The site‐specificity of ethnic differences in SSIp might suggest an interaction between biomechanical effects and other environmental factors. SSIp has been experimentally validated in animals and in humans.^48^, ^49^, ^50^ However, it must be interpreted with caution in children and adolescents because it is affected by changes in bone shape, size, and density distribution. These changes may all be occurring simultaneously during adolescent growth, which might lead to an apparent reduction in SSIp even when bone size and average cortical BMD are increasing. For example, if there is both apposition and resorption at different locations around the periosteum, such that the shaft cross‐section becomes less circular, then this could contribute to reductions in SSIp. Finally, differences in bone shape may also be a component of ethnic differences in SSIp: for the same CSA and cortical BMD, a more circular bone would have greater SSIp.^51^

### Limitations

There are limitations in the scope of the analysis. Although the age scale was offset to account for differences in APHV, we did not correct for differences in the rate of development, meaning that 1 year of biological age was regarded as equal to 1 year of chronological age. It is possible that divergence or convergence in the trajectories of some skeletal outcomes might be due to differences in the rate of growth, even after accounting for differences in the timing of growth. Furthermore, alignment on APHV provides only a crude adjustment for maturity, in part because anatomical regions differ in the timing of peak growth, with leg length preceding growth of the trunk.^52^, ^53^ APHV was used because it can be applied to both boys and girls and because a simple offset of the age scale retains the interpretation of slopes as annual changes in the outcome of interest. Another limitation is the change in scan protocol, with alterations in voxel size during the 10 years of follow‐up. We have adjusted for this as far as possible. Finally, in most cases, the model‐implied long‐term CVs are substantially greater than published short‐term CVs.^30^ However, long‐term precision of cortical BMD was found to be good, with CVs of less than 1.4% at the radius and less than 0.9% tibia.

## Conclusion

We have identified differences in the pattern of skeletal growth and development between black and white South Africans from the Birth to Twenty cohort, despite adjustment for known differences in maturational timing. This work gives new insight into the possible etiology of childhood fractures, which occur most commonly at peripheral sites. There were site‐specific differences both in absolute terms (at specific biological ages), and in the trajectories of development across biological ages. We did not adjust the trajectories for concurrent measures of height, weight, BMI, or body composition, all of which would also have been changing with biological age. Differences in CSA might be partially explained by anthropometric differences, but the site‐specificity of the skeletal differences suggests that these differences cannot be simple scaling effects. Furthermore, such adjustments may have obscured some of the differences we have identified, without necessarily providing any benefit for the interpretation of the results. However, any future investigation of potential environmental mechanisms must take anthropometric differences into account. For example, differences in nutrition have been reported in this cohort,^54^ and white children were more physically active and more likely to take part in formal sports classes than black children.^55^, ^56^ Such factors may have direct effects on bone, as well as indirect effects via their influence on growth and body composition. Further investigation of environmental factors, including nutrition and physical activity, will be required to fully understand these patterns and to what extent they are inherent or how they reflect differences in environmental exposures, ultimately contributing to an understanding of how these differences translate to current and future fracture risk.

## Disclosures

KW received an honorarium from Abbot Nutrition. There are no other conflicts of interest to disclose
